# Grain Boundary Evolution of Cold-Rolled FePd Alloy during Recrystallization at Disordering Temperature

**DOI:** 10.3390/ma8063254

**Published:** 2015-06-04

**Authors:** Hung-Pin Lin, Delphic Chen, Jui-Chao Kuo

**Affiliations:** 1 Department of Materials Science and Engineering, National Cheng-Kung University, Tainan 70101, Taiwan; E-Mail: n5896115@mail.ncku.edu.tw; 2 Department of Materials and Optoelectronic Science, National Sun Yat-Sen University, Kaohsiung 80424, Taiwan; E-Mail: delphi.home@msa.hinet.net; 3 Research Center for Physical Properties and Microstructure of Metals, National Sun Yat-Sen University, Kaohsiung 80424, Taiwan

**Keywords:** kernel average misorientation (KAM), electron backscatter diffraction (EBSD), recrystallization, FePd, low angle boundary (LAGB)

## Abstract

In this study, the grain boundary character and texture of 50% and 90% cold-rolled FePd alloy was investigated during recrystallization at 700 °C. Electron backscatter diffraction (EBSD) measurements were performed on the rolling direction to normal direction section. Kernel average misorientation (KAM) calculated from EBSD measurements was employed to determine the recrystallization fraction. The Avrami exponent n of recrystallization is 1.9 and 4.9 for 50% and 90% cold rolling, respectively. The new formation of texture reveals random texture during the recrystallization process. As annealing time increased, the number of high angle boundary (HAGB) and coincidence site lattice (CSL) increased with consumption of low angle boundary (LAGB). In addition, possible transformations between different grain boundaries are observed here.

## 1. Introduction

FePd, FePt, and CoPt alloys with L1_0_-ordered structures present potential applications in advanced gas-turbine and combustion engines, permanent magnet micro-devices, and data storage media [1,2,3,4]. One important feature that these alloys should present is operability at temperatures higher than 400 °C [5]. The Curie temperature *T*_C_ of FePd alloys is higher than 490 °C [6], and high saturation induction *B*_S_ approximates 1.38 T [3]. Compared with those of FeCo alloys, the *T*_C_ and *B*_S_ of FePd alloys are lower. However, FePd alloys present better ductile and corrosion resistance properties [6].

After a brief discussion on magnetic properties, we focus on recrystallization texture behavior. Disordered FePd alloys, such as brass, silver, and austenitic stainless steels, have low stacking fault energy (SFE) [3]. Several frequent texture components are indicated by name, such as the “Brass” or “B” ({011} <211>), “Copper” or “C” ({21l} <111>), “S” ({123} <634>), “Goss” ({01l} <100>), and “Cube” components ({00l} <100>). Smallman and Green [7] reported {112} <110> and {111} <110> components in the surface texture during hot rolling for austenitic stainless steels. Recrystallized non-ferrous face-centered cubic metals and alloys with low stacking fault energy present the major texture of {113} <211> [7,8,9,10,11,12]. In 95% cold-rolled AISI 304L austenitic stainless steel, the prominent components are Goss, Copper, and Brass in the temperature range between 600 and 1000 °C [12]. At 530 °C, the recrystallization texture of FePd alloy reveals the evident components, {010} <11 0 1> and {054} <22 −4 5>, after 50% and 90% cold-rolling, respectively [13].

Brass-type recrystallization textures are related to the deformation textures of <111> rotations by approximately 30° or 35° [14]. The {113} <211> orientation can be derived from the Brass component by 40° <111> rotation [9,15]. The Copper component has recently been reported to decrease in favor of the Cube component at the beginning of recrystallization [16,17]. The Brass component is subsequently consumed by the growth of Cube component, but at a considerably lower rate because of its unfavorable orientation relationship [18]. The Copper component is more rapidly consumed than the S component in favor of Cube component formation, which indicates the preferential nucleation of the Cube component in the transition band that possibly developed within the Copper component [19].

In addition to discussions on recrystallization texture, grain boundary migration plays an important role in primary static recrystallization. It is well known that the recrystallization takes place not only through the motion of high-angle grain boundaries [18] but also by that of low-angle grain boundaries [20,21,22,23,24]. However, the influence of low angle grain boundaries is usually not taken into account in experiments on the recrystallization process.

In the present study, the electron backscatter diffraction (EBSD) technique was employed to investigate the relationship between recrystallization behavior and grain boundaries in cold-rolled FePd alloy. After 50% and 90% cold rolling, FePd alloy was annealed at the disordering temperature of 700 °C for different times. Then, EBSD measurements were performed on the transverse section (TD) of the specimens.

## 2. Experimental Setup

The detailed cold rolling procedure for FePd alloy has been described in an earlier study [25]. After 50% cold rolling, specimens were annealed at 700 °C for 1, 2, and 2880 min (48 h); the specimens were annealed for 10, 20, and 60 s for 90% cold rolling and directly quenched in ice water. Prior to EBSD measurements, the transverse sections (TD) of the specimens were mechanically polished and then electro-polished in the electrolyte by a 4:1 mixture of acetic and perchloric acids at a charge current of 0.5 mA and constant voltage of 25 V for 30 s.

Finally, EBSD measurements were performed on the rolling direction (RD) to the normal direction (ND) section of the specimens. Here, a field-emission electron microscope (7001F, JEOL, Tokyo, Japan) was used together with an EBSD system (EDAX/TSL Technology) with 20 kV at a 15 mm working distance and a sample tilt of 70°. Step sizes of 0.35 and 0.15 µm were selected for 50% and 90% cold rolling, respectively. In addition, OIM™ software was employed for texture and microstructure analysis after annealing.

## 3. Results and Discussion

### 3.1. Determination of Recrystallization Behavior

**Figure 1:**
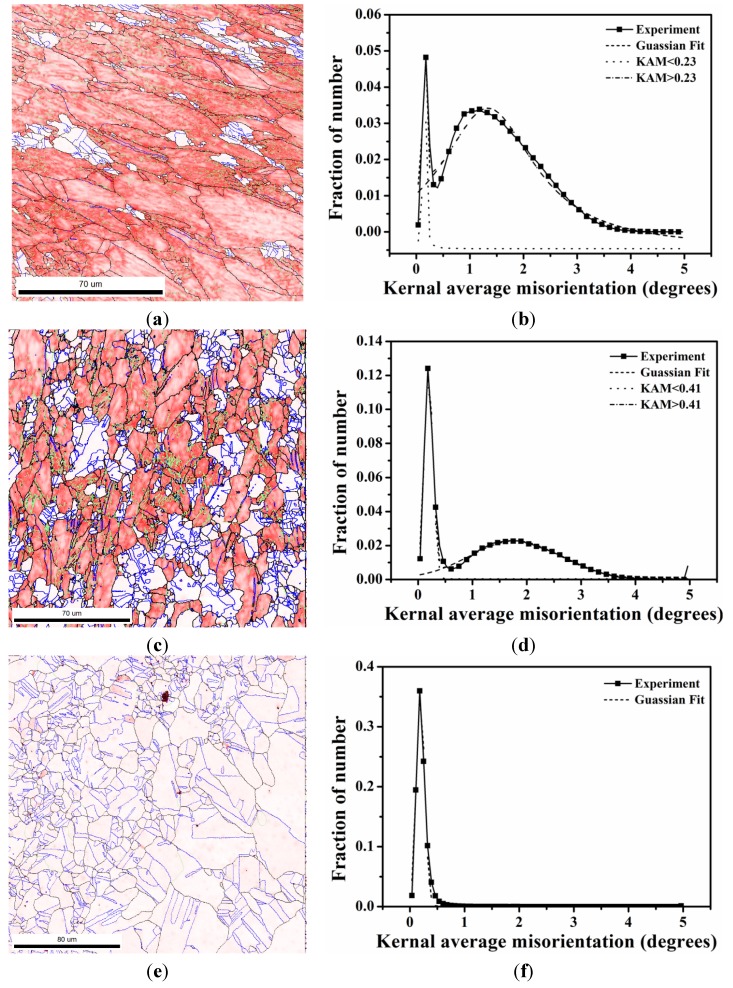
KAM maps after (**a**) 1 min; (**c**) 2 min; and (**e**) 48 h and KAM histogram after (**b**) 1 min; (**d**) 2 min; and (**f**) 48 h for 50% cold-rolled FePd alloy at 700 °C. (The KAM value ranges from 0° to 5°, corresponding to white and red. LAGB: Green, HAGB: Black and CSL: Blue).

**Figure 2:**
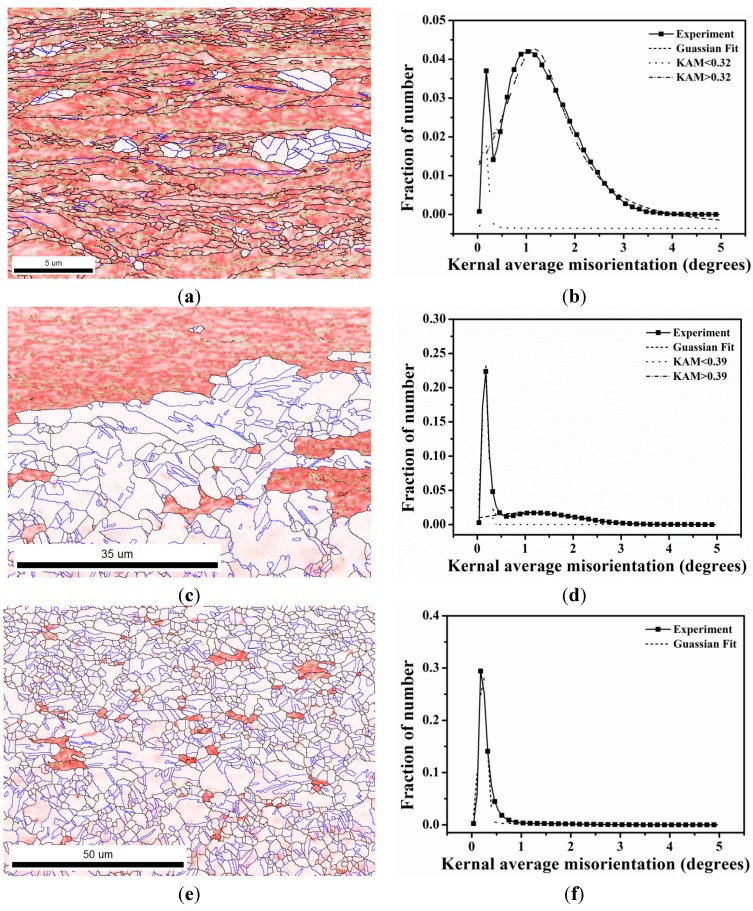
KAM maps after (**a**) 10 s; (**c**) 20 s; and (**e**) 60 s and KAM histogram after (**b**) 10 s; (**d**) 20 s; and (**f**) 60 s for 90% cold-rolled FePd alloy at 700 °C. The color coding is shown in Figure 1.

During recrystallization, the newly formed grains reveal a random texture. Kernel average misorientation (KAM) was used to represent local dislocation density distribution and the recrystallization fraction [13,26]. In principle, deformed grains have KAM > 1° because of their high dislocation density, whereas recrystallized grains have KAM < 1°. KAM maps are shown in Figure 1 and Figure 2 for 50% and 90% cold rolling after annealing at 700 °C, respectively. In this study, first, considering the case of 50% cold rolling, the KAM distributions are shown in Figure 1b,d,f after 1 and 2 min and 48 h annealing. Figure 1b,d show that the KAM distributions exhibit two peaks. The intersection point of two fitting curves for 1 and 2 min is at 0.23° and 0.41°. After 48 h annealing, only one peak is found in Figure 1f. Then, for 90% cold rolling, the KAM distributions are shown in Figure 2b,d,f for samples annealed at 700 °C for 10, 20, and 60 s. The intersection point of two fitting curves is at 0.32° and 0.39° after 10 and 20 s annealing, respectively. Therefore, the recrystallization fraction can be determined by calculating the area fraction of the fitting curves obtained from the KAM distributions shown in Figure 1b,d,f and Figure 2b,d,f. Thus, recrystallization fraction as a function of time can be estimated based on the Johnson-Mehl-Avrami-Kolmogorov (JMAK) Equation [27,28,29,30,31]. The JMAK Equation is given as follows:

(1) $$X=1-\exp(-kt^{n})$$

where *X* is the recrystallized fraction, *t* is time, *k* is the kinetic parameter, and *n* is the JMAK (or Avrami) exponent.

**Figure 3:**
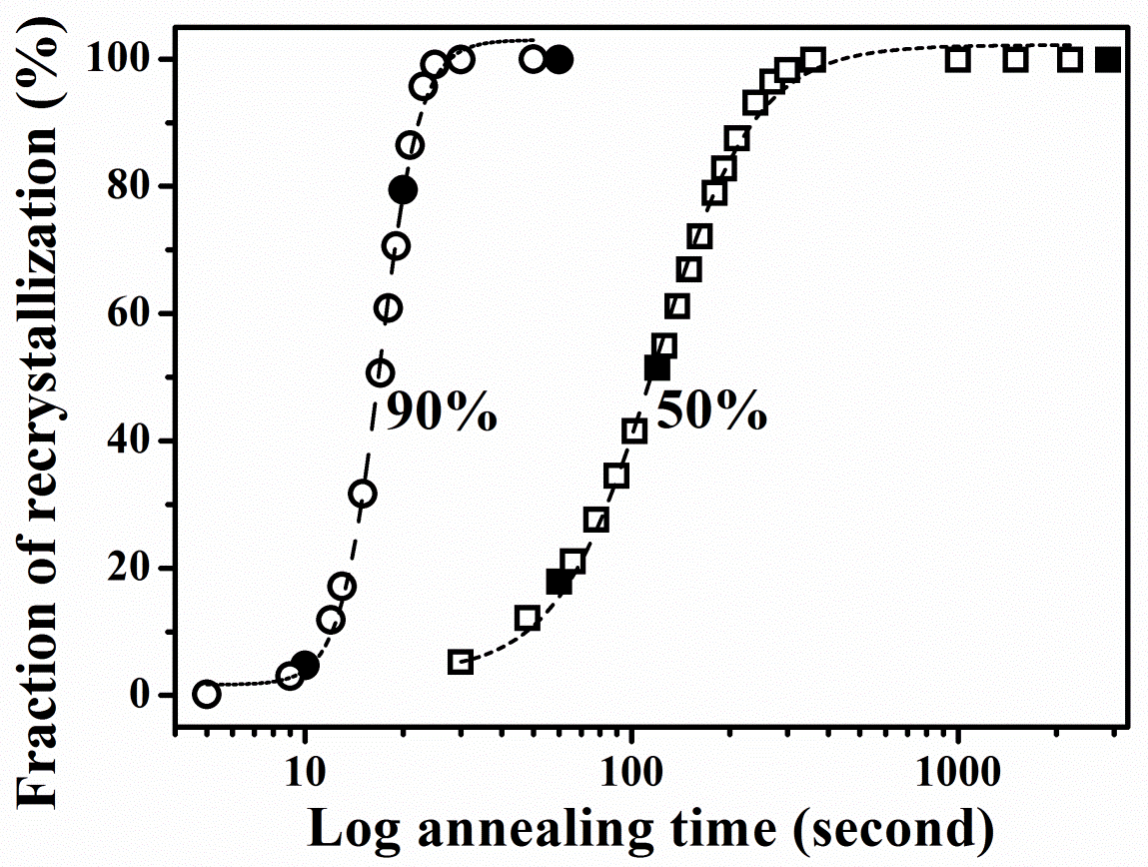
Recrystallization fraction during isothermal annealing for 50% and 90% cold-rolled FePd alloy at 700 °C. The solid symbol indicates the results obtained from EBSD, whereas the open symbol represents the predicted values obtained by using the JAMK equation.

According to the JMAK Equation in Figure 3 and Table 1, the Avrami exponent *n* is 1.9 and 4.9 for 50% and 90% cold rolling, respectively. Here, the *n* value of 1.9 approaches 2 [32], indicating one-dimensional growth, whereas 4.9, which approximates 5 and is larger than 4 [32], means three-dimensional growth at a constant nucleation rate. As reported, a value of *n* > 3 correlates with ideal recrystallization for homogeneous, constant-rate nucleation [33]. In addition, the twin fractions of 50% cold rolling are 17.4% and 40.6% after 1 and 2 min, whereas those of 90% cold rolling are 8.2% and 21.8% after 10 and 20 s, respectively. We observed that the twin fraction of 50% cold rolling is almost double that of 90% cold rolling. The formation of twins reduces the driving force for recrystallization, thus retarding the process [33,34]. This delay can be explained by the observed *n* value of 50% cold rolling, which is smaller than that of 90% cold rolling.

Considering the effect of ordering process on recrystallization in a previous study [14], the Avrami exponent *n* has the values of 0.7 and 1.1 for 50% and 90% cold rolling at 530 °C, respectively. In the case of 90% cold rolling, the twin fraction at 530 °C is smaller than that at 700 °C. Therefore, the *n* value at 530 °C should be larger than that at 700 °C according to retarding recrystallization because of twin formation. However, the *n* value at 530 °C is considerably smaller than that at 700 °C. Mao [35] *et al.* reported that for cold-rolled FeCo alloy, the ordered structure state can reduce grain boundary mobility and decrease the recrystallization speed in the ordering temperature region. Therefore, the above results indicate that the ordering process performs a considerably greater function than that of twin formation.

**Table 1:** Recrystallization fraction as a function of annealing time at 530 °C for 50% and 90% cold-rolled FePd alloy.

Annealing Time (h)	0.5	1	4	16	96	400
50% cold rolling	-	0%	-	33%	67%	100%
90% cold rolling	10%	26%	67%	100%	-	-

### 3.2. Effect of Grain Boundaries on Recrystallization

**Figure 4:**
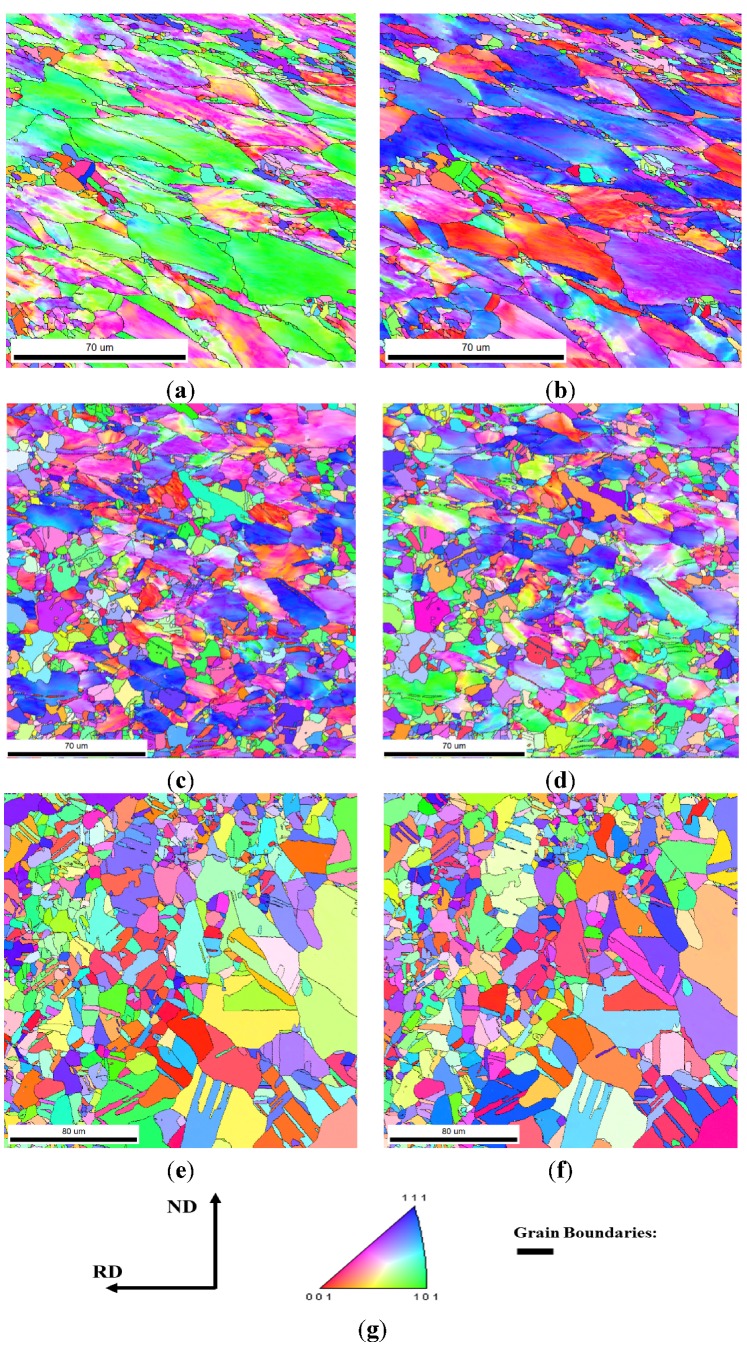
Inverse pole figure (IPF) maps of 50% cold-rolled FePd alloy after 700 °C annealing for (**a**,**b**) 1 min; (**c**,**d**) 2 min; and (**e**,**f**) 48 h. (**g**) The sample coordinates of ND and RD, and the corresponding color coding of the IPF in terms of ND.

**Figure 5:**
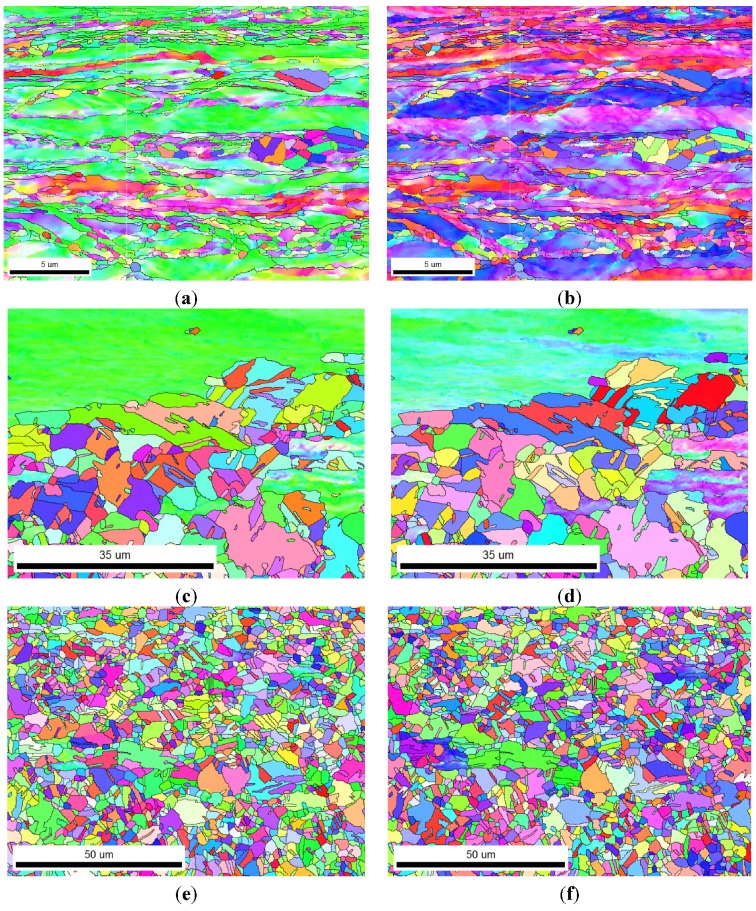
IPF maps of 90% cold-rolled FePd alloy after 700 °C annealing for (**a**,**b**) 10 s; (**c**,**d**) 20 s; and (**e**,**f**) 60 s. The corresponding color coding of the IPF is in terms of ND for (**a**,**c**,**e**) and RD for (**b**,**d**,**f**).

**Figure 6:**
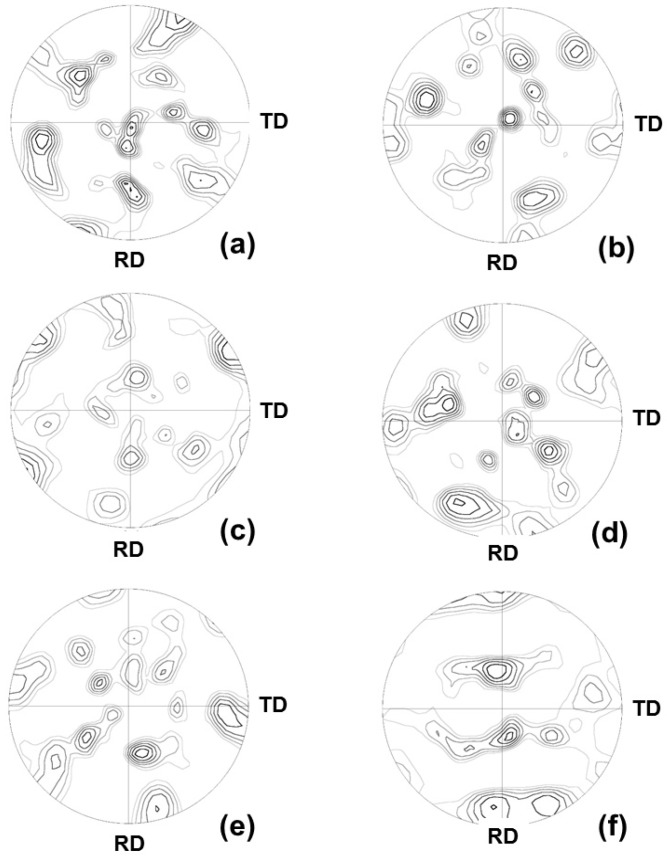
{111} pole figures of FePd alloy at 700 °C after (**a**) 1 min; (**c**) 2 min; and (**e**) 48 h for 50% cold rolling, and after (**b**) 10 s; (**d**) 20 s; and (**f**) 60 s for 90% cold rolling.

After discussing recrystallization behavior, we next analyzed the results of the recrystallization microstructure after 50% and 90% cold rolling. Figure 4 shows the inverse pole figure maps viewed from the ND and RD direction (called later as ND/RD-IPF) for 50% cold rolling at 700 °C for 1, 2, and 2880 min. Figure 5 shows the maps of 90% cold-rolled FePd alloy viewed from the ND and RD directions. To analyze the texture of recrystallization, {111} pole figures were recalculated from orientation distribution function (ODF), as shown in Figure 6. These {111} pole figure results after 50% and 90% deformation suggest that the newly formed grains during recrystallization reveal a random texture, which is in accordance with the inverse pole figure maps in Figure 1 and Figure 2. Therefore, the grain boundary is characterized in the following.

**Figure 7:**
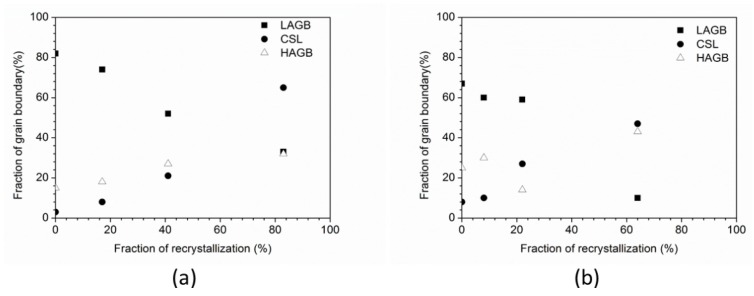
Fraction of grain boundaries as a function of recrystallization fraction for (**a**) 50% and (**b**) 90% cold-rolled FePd alloy at 700 °C. (LAGB: Low-angle boundary with misorientation between 5° and 15°, HAGB: High angle boundary with misorientation >15° and CSL: Coincidence site lattice).

We describe the character of grain boundaries according to the misorientation angle of two oriented grains. Here, three different types of grain boundaries are low-angle grain boundaries (called LAGBs) with misorientation of 5°–15°, high-angle grain boundaries (HAGBs) with misorientation >15° and special grain boundaries expressed in a coincidence site lattice (CSL) [32]. Figure 7a shows the grain boundary character distribution as a function of recrystallization fraction for 50% cold-rolled FePd alloy at 700 °C. It is found that the initial deformation microstructure of 50% deformation has 82% LAGB, 15% HAGB and 3% CSL. As increasing the annealing time from 1 min to 48 h corresponds to 17% to 83% recrystallization, the number fraction of LAGB is reduced but the number fractions of both HAGB and CSL are increased. This observation suggests the increase in boundaries of HAGB and CSL with consuming LAGB. In another case of 90% cold-rolled FePd alloy, it is observed to be the same as with 50%, but the difference between them lies in the ratio of HAGB to CSL.

**Figure 8:**
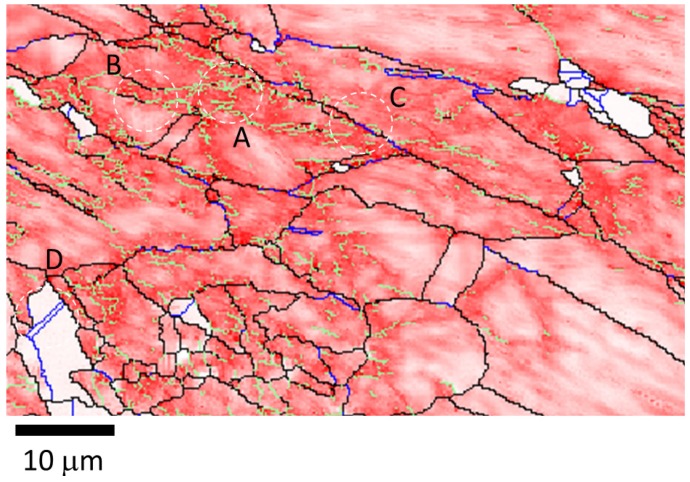
Magnification image of KAM map extracted from Figure 1a for 50% cold-rolled FePd alloy after 1 min annealing at 700 °C. The color code is shown in Figure 1.

**Figure 9:**
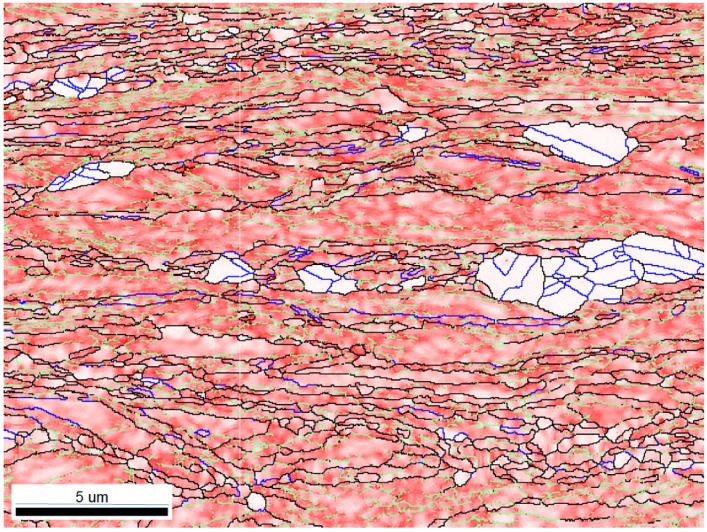
Magnification image of KAM map extracted from Figure 2a for 90% cold-rolled FePd alloy after 10 s annealing at 700 °C. The color code is shown in Figure 1.

**Figure 10:**
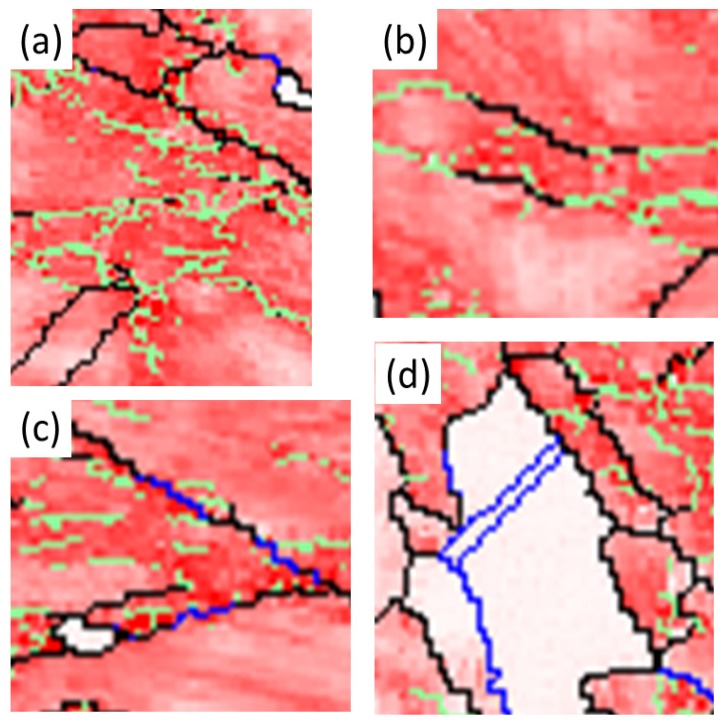
Magnification image of (**a**) A area with LAGBs; (**b**) B area with HAGB and LAGB; (**c**) C area with HAGB and CSL; and (**d**) D area with CSLs for 50% cold-rolled FePd alloy at 700 °C after 1 min. A, B, C and D symbols indicate the areas of image magnification marked in white dash lines in Figure 8.

The succession of grain boundaries indicates a change in boundaries at different stages of recrystallization as mentioned above. In order to reveal the partially recrystallized grain boundaries at the early stage, the KAM maps were overlapped with LAGB, HAGB and CSL boundaries in Figure 8 and Figure 9 for 50% and 90%, respectively. The kernel average misorientation map can be assumed to be a dislocation density distribution map in different grains [13,26]. After 50% and 90% deformation LAGB reveals random boundaries, but after one minute of annealing, some LAGBs can be retained at the early stage of recrystallization in Figure 10a. At this stage LAGB distribution reveals “regular” boundaries in Figure 10a which occur in the vicinity of high KAM, that is, regions with a high density of dislocations. The development of LAGB distribution from “random” to “regular” can be explained with the mechanisms of thermally activated glide and cross-slip after a short annealing time. These mechanisms lead to a decrease in dislocation density and the LAGB number.

**Figure 11:**
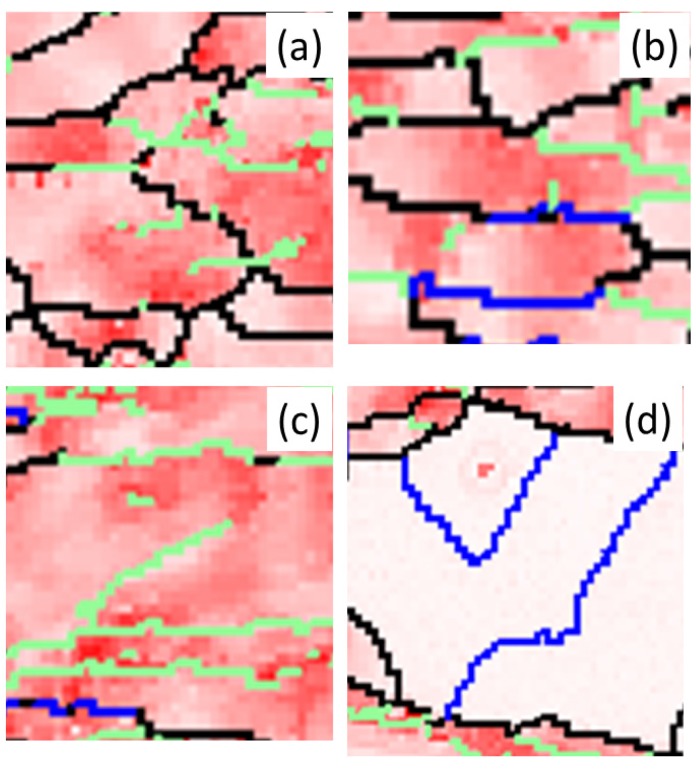
Magnification image of (**a**) A area with LAGBs; (**b**) B area with HAGB and LAGB; (**c**) C area with HAGB and CSL; and (**d**) D area with CSLs for 90% cold-rolled FePd alloy at 700 °C after 1 min. A, B,C and D symbols indicate the areas of image magnification marked in white dash lines in Figure 8.

In addition to the formation of regular LAGBs, two combination grain boundaries are observed; one is LAGB/HAGB, and the other CSL/HAGB, as shown in Figure 10b,c. Both types of combination grain boundaries also have high KAM as mentioned in LAGBs. In the case of 50% deformation with one minute annealing, KAM > 0.23 is assumed in deformed state and KAM < 0.23 in recrystallized state in Figure 1b. These boundaries of LAGB, LAGB/HAGB and CSL/HAGB have KAM > 0.23 belonging to the deformed state. LAGB boundaries occur firstly in deformed state and can remain in the early stage of recrystallization. However, CSL boundaries with KAM < 0.23 in Figure 10d are finally formed during recrystallization. Between LAGB and CSL there is the transition state of LAGB/HAGB and CSL/HAGB. LAGB/HAGB and CSL/HAGB boundaries with KAM > 0.23 suggest that both of them are not fully recrystallized as the case of CSL, that is, the transition state. It is usually assumed that low-angle grain boundaries are immobile. However, Winning and Raabe [36] reported that low-angle grain boundaries can move during recrystallization. This finding can be explained by the transformation from LAGBs to the transition state of LAGB/HAGB. After the LAGB transformation, HAGBs can be transformed to the transition state of CSL/HAGB due the low interface energy of CSL. These observations are also found in 90% deformation in Figure 11. Therefore, we can conclude that during the recrystallization process, grain boundaries can transform from LAGB through HAGB and to CSL boundaries.

## 4. Conclusions

After 50% and 90% reduction in FePd alloy, the exponent *n* of 1.9 and 4.9 indicate one- and three-dimensional growth at constant-rate nucleation, respectively. Compared to 90% reduction, the smaller *n* value of 50% reduction is due to its higher twin fraction. During recrystallization, HAGB and CSL increase with the consumption of LAGB at first, and the later increase in CSL is due to a decrease in HAGB. Therefore, we observed that a change in grain boundaries occurs from LAGB through HAGB to CSL during recrystallization for 50% and 90% reduction. Furthermore, the newly formed grains reveal a random texture at the early stage of recrystallization for 50% and 90% reduction.
